# Meetings of the Bristol Medico-Chirurgical Society

**Published:** 1893-06

**Authors:** 


### Bristol Medico-Chirurgical Society.

*April 12th*, 1893.

Dr. E. Markham Skerritt, President, in the Chair.

Dr. Armand Ruffer gave an account of some recent researches by himself and Mr. H. G. Plimmer, on micro-organisms found in cancer, and exhibited numerous photographs with the oxy-hydrogen lantern, and microscopic slides. The parasites to which he referred had been found in various carcinomata, but he limited himself to a short description of those seen in cancer of the breast. The organism often appeared in the nucleus of the cell; it was a small, round body, staining with hæmatoxylin in osmic acid preparations. At first it presented no structure, but later a small nucleus and capsule became apparent, and a peripheral radiation. It ultimately slipped from the nucleus into the surrounding protoplasm. As the parasite grew, it often became granular, and contained peculiar chromatin bodies; the capsule had a well-marked double contour. Such bodies could be plainly seen in fresh specimens kept on the warm stage, as well as in stained cover-glass preparations fixed with the vapour of hydrocyanic acid, and stained with Biondi's reagent. The mode of reproduction was by single fission, the nucleus first dividing; but division into eight, sixteen, or more might occur. Whether these young parasites were of the nature of spores it was impossible at present to say. Outside the body these organisms appeared to resist putrefaction much longer than the tissues in which they were found. Lastly, Dr. Ruffer drew attention to the fact that it was useless to search for these parasites with old methods, and that it was only by using the best fixing and staining reagents that satisfactory results could be obtained. At the conclusion of the meeting a vote of thanks to Dr. Ruffer and Mr. Plimmer was carried by acclamation.

---

*May 10th*, 1893.

Dr. E. Markham Skerritt, President, in the Chair.

Dr. Edgeworth exhibited a child with bilateral facial paralysis dating from birth. Electrical stimulation gave no results, but there was slight voluntary movement at the angles of the mouth. No sign of injury to the skull could be detected. It was thought the condition might be due to the application of forceps in delivery.

Mr. C. A. Morton showed a child with genu varum, following a fracture of the femur a short distance above the knee. There was marked elongation of the external condyle, and it was pointed out that the pathology of this case was similar to that of knock-knee with enlargement of the *internal* condyle. Mr. Morton also exhibited an infant with talipes calcaneo-valgus, associated with intra-uterine bending of the bones of the leg; specimens of Fallopian tubes, uterus and vagina of child affected with tuberculosis; lymphatic nævus of tongue; and sarcoma of peritoneum causing fatal perforation.

Dr. J. E. Shaw and Mr. Paul Bush brought before the Society a case of lesion of the cauda equina. This will be published *in extenso* in the *Journal.*

Mr. Greig Smith showed the Fallopian tubes, ovaries, and a small uterine fibroid, removed from a lady aged 23. Portions of these structures were markedly calcified.

Mr. Munro Smith and Mr. C. A. Morton showed microscopic sections of two complex tumours—one an adenomatous growth from the soft palate, the other from the eyebrow. Both were thought to be congenital.

Dr. Michell Clarke showed hydatid cysts from the brain, which hollowed out a large portion of the cortex and gave rise to Jacksonian convulsions, &c.; also various microscopic sections of the spinal cord from cases of progressive muscular atrophy and ascending degeneration.

Dr. Watson Williams gave a demonstration on some interesting laryngeal cases.

G. Munro Smith, *Hon. Sec.*

